# Consensus document on palliative care in cardiorenal patients

**DOI:** 10.3389/fcvm.2023.1225823

**Published:** 2023-12-19

**Authors:** Clara Bonanad, Juan M. Buades, Juan Pablo Leiva, Rafael De la Espriella, Marta Cobo Marcos, Julio Núñez, Helena García-Llana, Lorenzo Facila, Rosa Sánchez, Laura Rodríguez-Osorio, Alberto Alonso-Babarro, Borja Quiroga, Daznia Bompart Berroteran, Carmen Rodríguez, Daniela Maidana, Javier Díez

**Affiliations:** ^1^ Cardiology Department, Hospital Clínico Universitario de Valencia, Valencia, Spain; ^2^ Biomedical Research Institute (INCLIVA), Valencia, Spain; ^3^ Center for Network Biomedical Research of Cardiovascular Diseases (CIBERCV), Carlos III Institute of Health, Madrid, Spain; ^4^ Nephrology Department, Hospital Universitario Son Llàtzer, Palma de Mallorca, Spain; ^5^ Institute for Health Research of the Balearic Islands (IdISBa), Palma de Mallorca, Spain; ^6^ Support and Palliative Care Team, Hospital Manacor, Palma de Mallorca, Spain; ^7^ Cardiology Department, Hospital Puerta del Hierro, Madrid, Spain; ^8^ Universidad Internacional de La Rioja (UNIR), La Rioja, Spain; ^9^ Centro de Estudios Superiores Cardenal Cisneros, Universidad Pontifica de Comillas, Madrid, Spain; ^10^ Cardiology Department, Consorcio Hospital General de Valencia, Valencia, Spain; ^11^ Nephrology Department, Hospital Universitario General de Villalba, Madrid, Spain; ^12^ Support and Palliative Care Team, Hospital Universitario la Paz-(IdiPAZ), Madrid, Spain; ^13^ Nephrology Department, Hospital Universitario de la Princesa, RICORS2040, Madrid, Spain; ^14^ Nephrology Department, Hospital Universitario Central de Asturias, Oviedo, Spain; ^15^ Center for Applied Medical Research (CIMA), and School of Medicine, Universidad de Navarra, Pamplona, Spain

**Keywords:** chronic heart failure, chronic kidney disease, cardiorenal patients, palliative care, cardiovascular medicine

## Abstract

There is an unmet need to create consensus documents on the management of cardiorenal patients since, due to the aging of the population and the rise of both pathologies, these patients are becoming more prevalent in daily clinical practice. Chronic kidney disease coexists in up to 40%–50% of patients with chronic heart failure cases. There have yet to be consensus documents on how to approach palliative care in cardiorenal patients. There are guidelines for patients with heart failure and chronic kidney disease separately, but they do not specifically address patients with concomitant heart failure and kidney disease. For this reason, our document includes experts from different specialties, who will not only address the justification of palliative care in cardiorenal patients but also how to identify this patient profile, the shared planning of their care, as well as knowledge of their trajectory and the palliative patient management both in the drugs that will help us control symptoms and in advanced measures. Dialysis and its different types will also be addressed, as palliative measures and when the decision to continue or not perform them could be considered. Finally, the psychosocial approach and adapted pharmacotherapy will be discussed.

## 1. Reason for palliative care in cardiorenal patients

The World Health Organization defines palliative care (PC) as actions to alleviate severe health-related suffering (1). However, only about 14% of people needing PC worldwide currently receive it (2). Cardiorenal syndrome (CRS) involves five types of disorders that affect both the heart and kidneys, in which dysfunction in one organ may induce dysfunction in the other (3, 4). However, due to increasing complexity and risk factors, epidemiological, diagnostic, preventive, and therapeutic aspects need to be considered beyond the limited context of organ dysfunction. Current advances have challenged the classification of the CRS.

Therefore, the time has come to transition from a taxonomic approach, which categorizes kidney and heart diseases separately, to a more comprehensive approach that recognizes the interplay between these two conditions. This broader approach is based on the concept of cardiorenal (or renocardiac) patients. These patients are primarily diagnosed with either heart disease or kidney disease, but as their clinical course progresses, they develop other conditions as well. From this perspective, a multidisciplinary group of general practitioners, palliative physicians, cardiologists, and nephrologists from hospitals in different parts of Spain considered it essential to prepare this consensus document on PC in patients with concurrent chronic heart disease and chronic kidney disease (CKD).

Patients with chronic heart failure (CHF) and CKD often require PC since these are chronic conditions whose symptoms are related to volume overload, exercise intolerance, pain, and depression (5–8). The interlinked cycle of heart and kidney failure, competing and conflicting treatment strategies, and complex care increase the risk of poor quality of life and mortality. The prevalence of both diseases is expected to increase worldwide in the coming years, and nowadays, a significant proportion of healthcare spending is spent on patients with both diseases. In addition, decedents with both conditions require more intensive end-of-life care and shared decision-making processes than those with only one condition (9–11).

**Figure 1:**
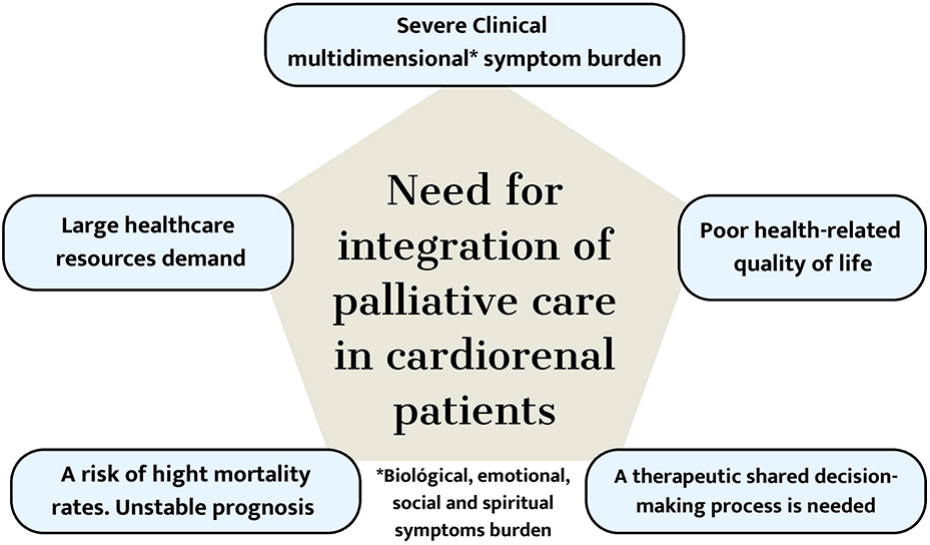
The Pentagon needs to be considered for integrating palliative care into the care of cardiorenal patients.

Patients with CHF and CKD have unmet medical needs in PC due to symptom burden, poor quality of life, high mortality, and healthcare resource demand, among other reasons. It is suggested that concurrent involvement with this modality of care is suggested, and needs-based referral criteria can foster early integration with optimal cardiology and nephrology care. These criteria apply to general cardiologists and nephrologists rather than advanced specialists and do not require patients to be in advanced stages before intervention (Figure 1).

To provide optimal care for cardiorenal patients, integrating renal, cardiac, and PC is crucial. Considering resource availability and allocation, an episodic model of short-term integrated palliative and supportive care (SIPS) may be appropriate for the up-and-down trajectory of the cardiorenal disease. This model was initially developed for geriatric patients, but a similar delivery schedule and integrated work processes between specialists may benefit cardiorenal patients. Its “episodic” attribute accommodates brief, prolonged, or returning PC follow-up, facilitating care integration per the patient's evolution.

CRS as an entity is relatively new and complex. Therefore, experience is needed based on the specific skills, knowledge, and attitudes that professionals must acquire, as well as logistical and administrative actions by organizations. All the considerations mentioned above could be addressed if we aim to provide the best quality of care for people with cardiorenal disease (12).

## 2. Methodology

The need for a global view of the management of patients with advanced cardiorenal disease in need of PC was identified by an emerging geriatric research group led by an expert in the field since 2016 who served as chair of the Geriatric Cardiology Section (2019–2021). They contacted members of the “Renal Supportive and Palliative Care” (that included Palliative Care Specialists) and “Cardiorenal Medicine” working groups of the Spanish Society of Nephrology (SEN) and members of the Spanish Planning Association for Care (AEPCA) to design this document.

A writing group was commissioned to review the current literature and to develop an expert-based consensus summary on CRS and PC. Members of the writing group were chosen for their expertise in HF, CKD, PC, shared care planning (SCP), and therapeutic strategies in managing CRS. The writing group held a series of teleconferences and web-based communications from June 2022 to November 2023. A manuscript outline was developed on the initial conference call, with individual section reviews being assigned to authors based on their expertise. All authors had continuous access to the working document to provide input, and each section editor provided critical review and periodical revisions.

The writing group used the main PubMed (1966–present), which was limited to human subjects and the English and Spanish languages. Related article searches were conducted in PubMed and Cochrane Database to find relevant articles. In addition, writing group members recommended articles outside the scope of the formal searches. Key relevant search words and Medical Subject Heading descriptors included kidney disease, renal insufficiency, chronic renal/chronic kidney, end-stage renal or end-stage kidney disease, congestive/myocardial/heart failure, cardiorenal, PC, and shared decision support. Key search abbreviations included CRS, CKD, HF, and PC. Finally, findings from conference proceedings, medical textbooks, and relevant online data sources were reviewed. Specific topics within the consensus document may have been reviewed in other clinical practice guidelines and scientific statements published by other working groups. When appropriate, these relevant guidelines have been referenced without the need to reiterate recommendations contained in those guidelines or statements.

## 3. How do we identify patients with cardiorenal disease needing palliative care?

### 3.1. Comprehensive geriatric assessment in the cardiac patient focused on HF

The improvement in survival rates worldwide presents new challenges in the fields of cardiology, nephrology, and clinical medicine in general. Elderly patients have unique characteristics that require a comprehensive geriatric assessment due to atypical disease presentations, susceptibility to polypharmacy, heterogeneity in aging, and multiple comorbidities (13).

Comprehensive geriatric assessment (CGA) is a diagnostic process that evaluates the cognitive, functional, and social conditions of elderly patients in a systematic, multidimensional, and dynamic manner. CGA is the most widely validated assessment in clinical practice that allows individualized diagnostic and therapeutic plans to be carried out to obtain the best results (1, 14). CGA requires time and specialized personnel. Scales like the Multidimensional Prognostic Index (MPI) and Edmonton Frail Scale (EFS) allow for an abbreviated assessment by non-geriatric physicians (15). The application of CGA is associated with lower mortality and improved survival rates (16). Furthermore, it is also associated with improved diagnostic accuracy, patient quality of life, quality of care, use of hospital resources, institutionalization, and costs.

**Figure 2:**
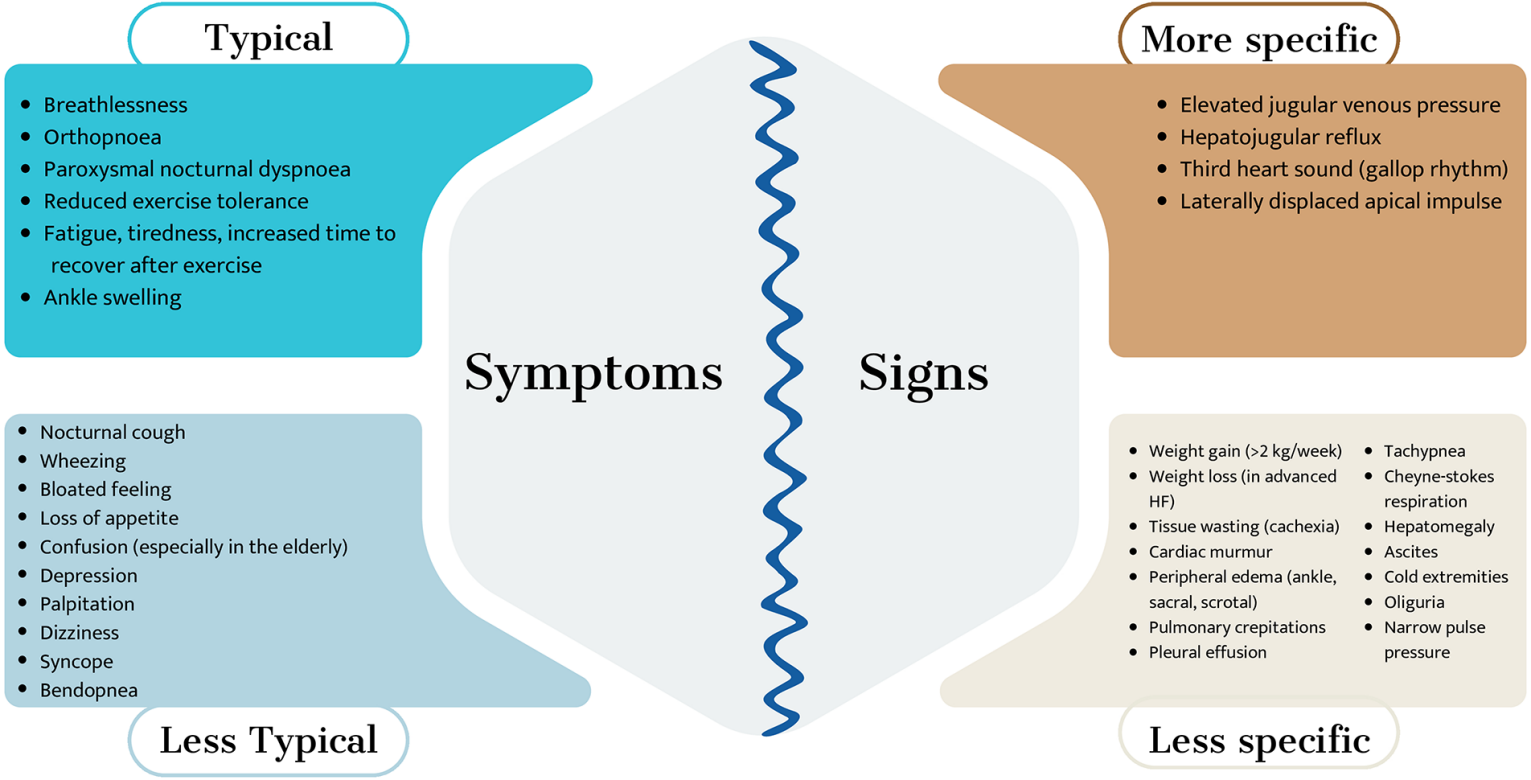
Symptoms and signs of heart failure.

HF is a clinical syndrome that mainly affects the elderly population and is the leading cause of hospitalization among those over 65 years of age (17). Hospitalization for HF is associated with increased comorbidity, requiring specific strategies aimed at this population. HF is characterized by the presence of cardinal symptoms and signs and results from a structural and/or functional cardiac abnormality, leading to decreased cardiac output and/or elevated filling pressures at rest or during exercise (18) (Figure 2).

Clinical guidelines use three categories to assess left ventricular ejection function: HF with reduced LVEF, HF with mildly reduced LVEF, and HF with preserved LVEF. Structural heart disease or diastolic dysfunction must be demonstrated in the last two cases, in addition to elevated natriuretic peptides. In HFrEF, treatment with neurohormonal blockers should be started with caution in elderly patients. Identifying the cause of cardiac dysfunction is crucial for determining appropriate treatment and deciding when non-intervention and palliative care are necessary. Frailty is a state of vulnerability due to a stressful situation associated with an increased likelihood of adverse outcomes. There are two approaches to characterizing frailty:

• On the one hand, the physical frailty phenotype considers frailty as a state before the disability. It relies primarily on finger grip strength tests and the gait speed test.
• On the other hand, frailty is defined as a set of deficits (multidimensional frailty), including comorbidities, disability, symptoms, and laboratory data. It is a situation with a high risk of adverse outcomes.

Frailty is more common in women than in men and increases with age, especially among patients with cardiovascular disease. Its prevalence ranges from 4% to 14% in older adults without disabilities, up to 21% in Spain. Among patients with acute HF, the frequency of frailty ranges between 50%–70%, with adverse short and long-term outcomes. Frailty is potentially reversible, so early detection is crucial (19–22). The major drawback of assessing frailty in the acute phase arises from the difficulty of applying scales that include physical performance tests; they also require time and an adequate environment.

In acute situations, scales based on self-referenced questions or clinical judgment of healthcare personnel are helpful. A self-referenced question scale based on Fried's criteria can identify high-risk acute HF patients (23). The FRAIL scale and other scales based on impairment accumulation are also worthwhile. After symptom stabilization, measuring frailty with Fried's phenotype is feasible. Global scales such as the Clinical Frailty Scale or MPI may be useful in the advanced stages of the disease. Low physical activity and slow gait speed are independent predictors of death and rehospitalization for HF, and gait speed is not included in usual scores (24–26).

Frailty is not the same as disability. It is a stage prior to disability, which is associated with increased mortality, falls, hospitalization, functional deterioration, and institutionalization. For this reason, it should be assessed before disability occurs.

Although these patients are at greater risk of complications, the existence of frailty without an associated advanced disability does not in itself contraindicate any intervention. Decisions should be made in a multidisciplinary manner, requiring close monitoring and early intervention of those aspects that are modifiable.

### 3.2. Indications for PC in the end-stage of HF

Persistent symptoms characterize advanced HF despite maximal therapy, and its prevalence is increasing due to various factors (7). The prognosis is poor, with a one-year mortality rate ranging from 25% to 75%, and many patients die of cardiac causes unaware of their disease's prognosis (27). PC in HF patients improves the quality of life, depression, anxiety, and spirituality (28). Specific clinical, cardiac, and analytical markers were shown to be associated with poor HF prognosis (1).

**Figure 3:**
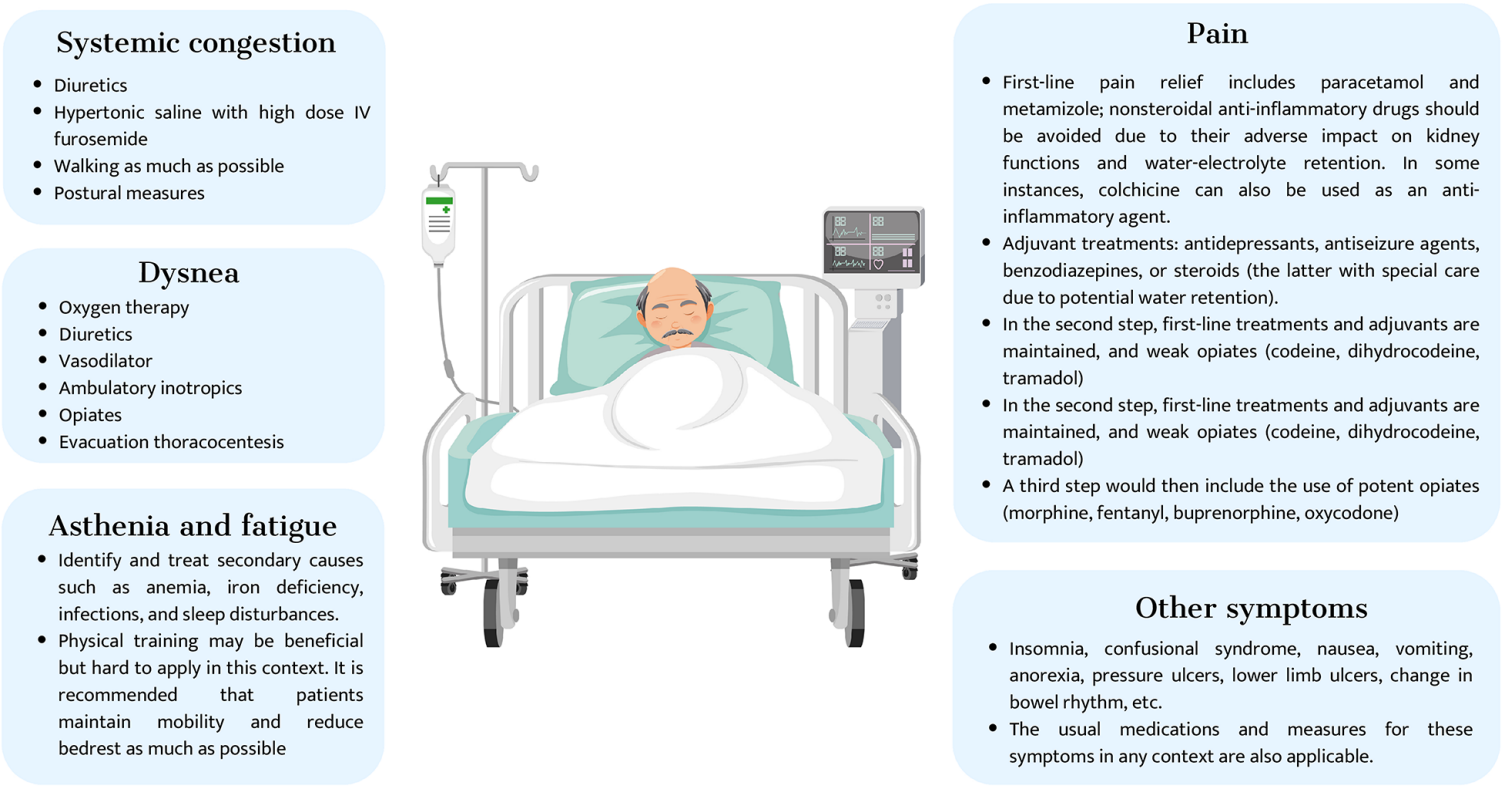
Interdisciplinary palliative care intervention in advanced heart failure.

When to consider palliative care? An interdisciplinary intervention in advanced HF patients consistently showed more significant benefits in quality of life, anxiety, depression, and spiritual well-being compared to usual care (28). Ideally, palliative care should be introduced early in the disease trajectory and increased as the disease progresses. Discussions about poor prognosis, the need for palliative care, and care goals can begin at annual HF review visits in less advanced stages or after each significant health-related event in more advanced stages (Figure 3).

Symptom control constitutes one of the essential pillars of PC. It enables relief of suffering, avoids futile life-prolonging measures, and promotes patient and family adaptation (Figure 3).

Bases of palliative care management in patients with HF:

• The treatment unit comprises the patient-family binomial (or inner circle). The family, as well as the patient, needs specific accompaniment and education for care and decision-making. Remember that the patient's fundamental nucleus of support resides both at home and in the hospital.
• Comprehensive and individualized care. To perform a multidimensional assessment, physical, emotional, social, and spiritual aspects must be considered. For this purpose, health personnel must be attentive with a comprehensive view of the complexity of the patient. Health professionals often fail to determine the proximity of a patient's death, which results in poor patient management. The comprehensive evaluation of the patient allows the planning of multidisciplinary and anticipatory treatments in which the response can be monitored and readjusted if necessary.
• Active therapeutic attitude: Therapeutic success means benefiting the patient, including device review (e.g., implantable cardioverter defibrillator) and frequent medication review to avoid events due to inappropriate handling by polypharmacy. Oral medication is preferred for analgesics, with subcutaneous administration as an alternative, depending on the medication. Conduct a thorough symptom anamnesis and identify and treat reversible causes, such as frailty. Review and adjust therapeutic objectives based on disease progression and establish advance directives (29).
• Communication: Encourage respect, comfort, and fluid communication between the patient, the family, and the healthcare team. This relationship promotes patient autonomy and shared decision-making. Take an empathic attitude, listen to the patient, ask for their opinion, be patient, and explain as often as necessary. Decision-making is always based on the patient's autonomy and dignity.

**Figure 4:**
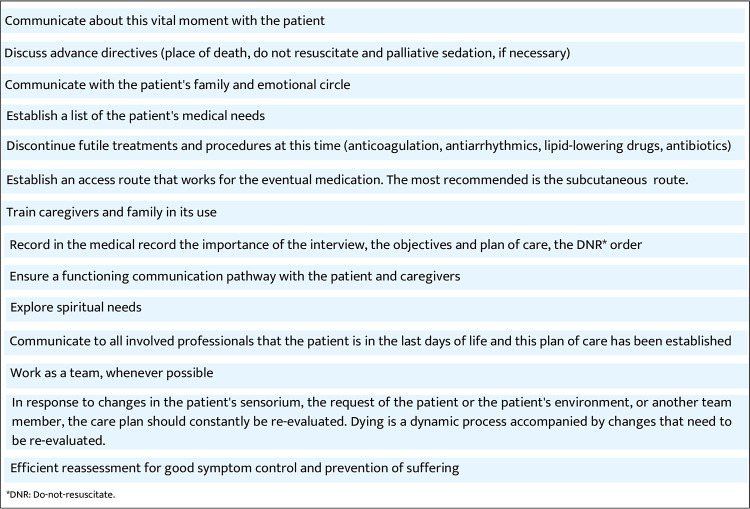
End-of-life care.

Finally, it should be remembered that end-of-life care is only one part of palliative care and that multidisciplinary teams obtain the best results. Hospice care focuses on providing comprehensive and supportive care to individuals who are in the end stage of a terminal illness and have a limited life expectancy (typically six months or less). The primary goal is to provide comfort and care rather than cure. On the other hand, palliative care can be offered at any stage of a severe illness, regardless of life expectancy. The main objective of palliative care is to control symptoms, alleviate pain, and improve the patient's quality of life (30) (Figure 4).

### 3.3. Indications for PC in cardiorenal patient

**Figure 5:**
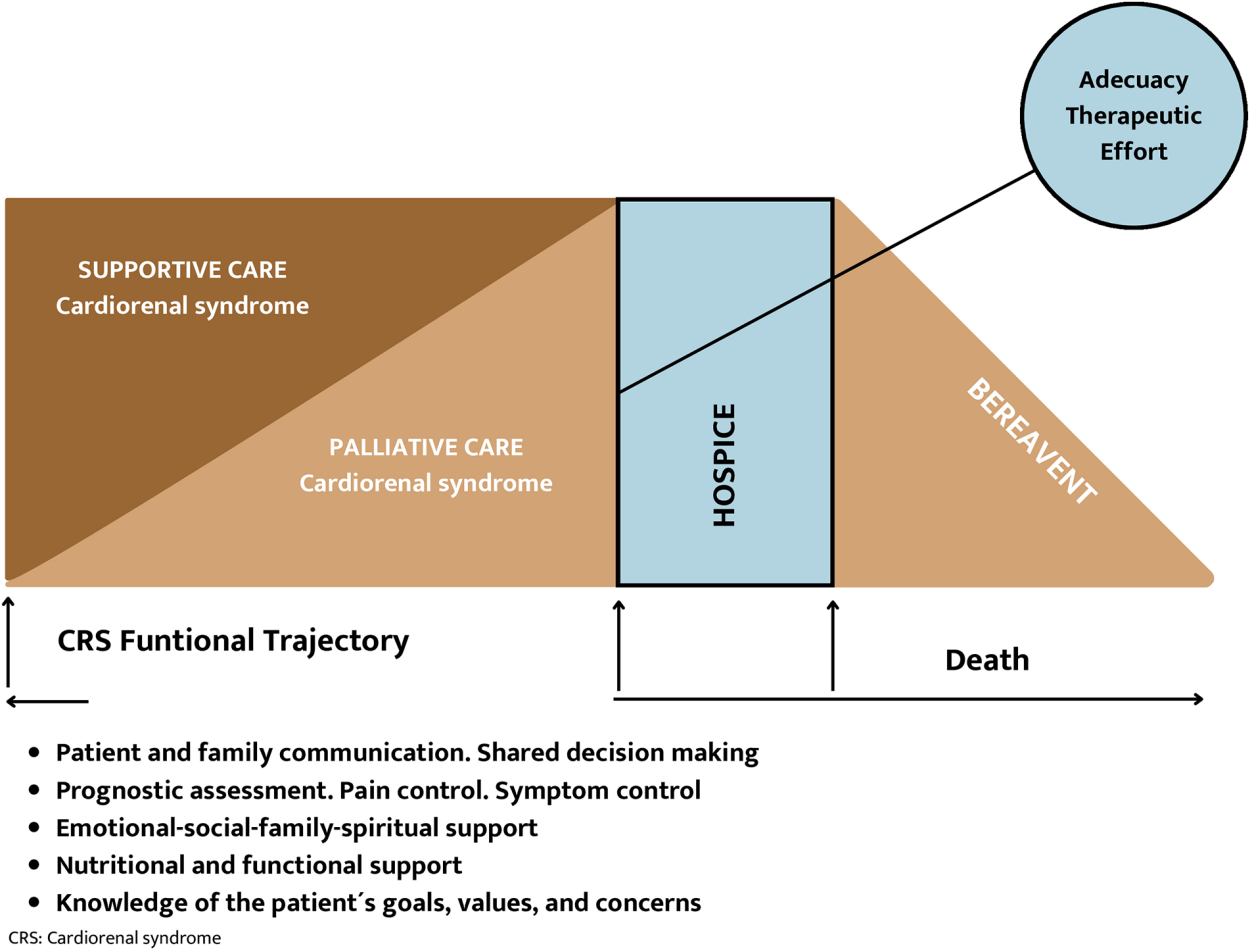
Conceptual framework of palliative care in cardiorenal syndrome.

Patients with advanced HF and CKD (cardiorenal patients) may face similar considerations as other patients with advanced HF but with some specific factors. Due to their high comorbidity, symptom burden, and higher morbidity and mortality rates than the general population, cardiorenal patients are often suitable for PC. The palliative needs of cardiorenal patients and their families are like those of patients with progressive and incurable non-malignant diseases, such as cancer. Including PC in the care of cardiorenal patients can improve their quality of life, facilitate communication between patients and healthcare providers, optimize assessment and symptom management, promote functionality, provide psychosocial support, and help achieve adequate care coordination to reduce hospital admissions (31–33). Introducing PC programs would enable a smooth transition from active treatment to PC and facilitate the setting of care objectives that align with the patient's prognosis. These goals should prioritize the management of symptoms and respect the patient's preferences. They should be accessible from diagnosis to the end of life, and a multidisciplinary approach with effective coordination between specialists, primary care, and PC physicians is crucial (34) [Figure 5 (35)].

#### 3.3.1. Roadmap for the development of PC programs

Implementing PC programs in CRS begins by detecting susceptible patients and establishing care objectives based on prognosis.

**Figure 6:**
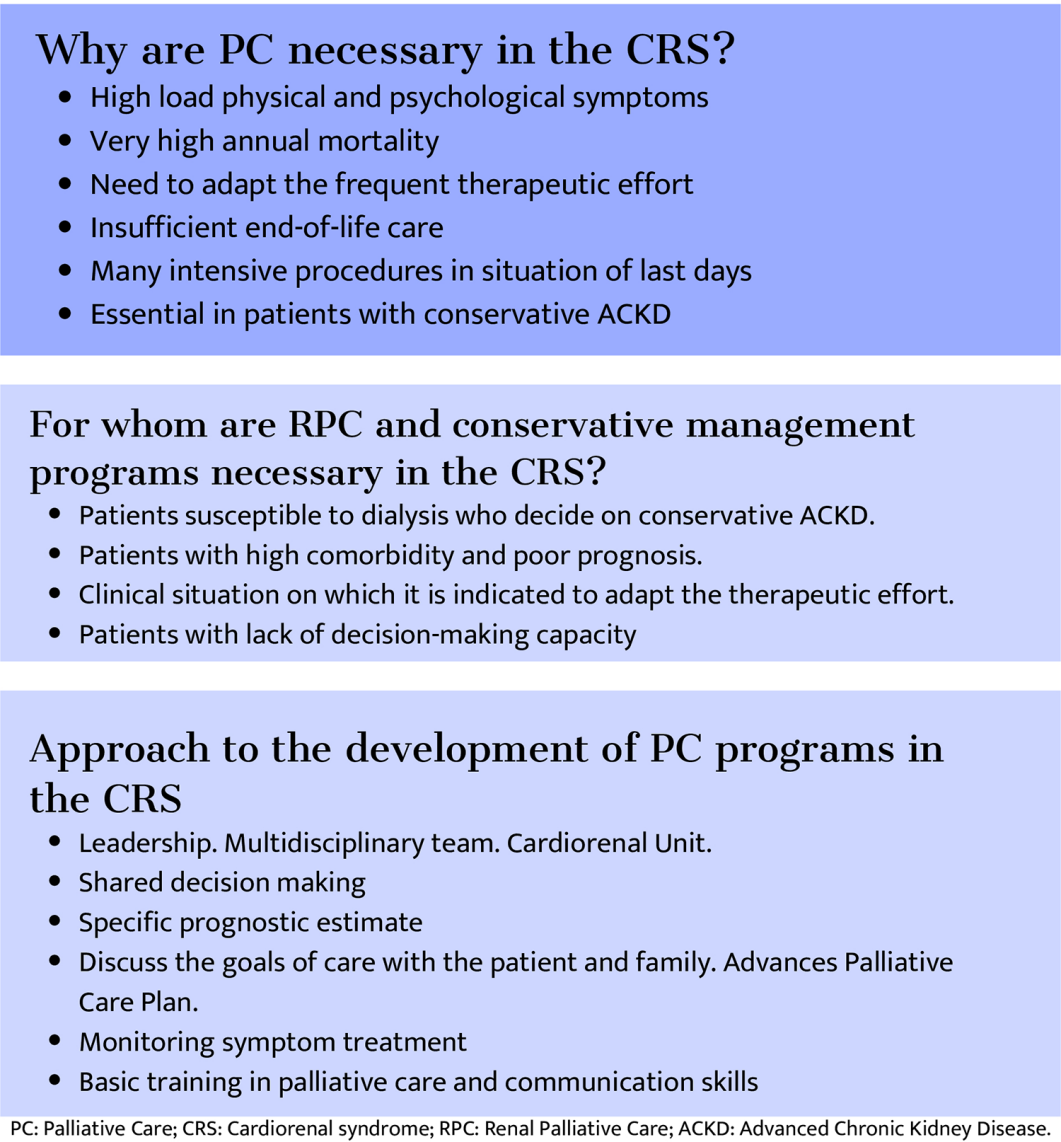
Roadmap for the development of supportive and palliative care programs in cardiorenal syndrome.

Adequate resources must be available to ensure conservative Advanced Chronic Kidney Disease (ACKD) care that facilitates ethically acceptable decision-making and discussions with the patient and family about palliative treatments. Hence, the growing interest and need for including renal PCs in developing clinical guidelines already propose a roadmap on why and for whom renal PCs are necessary and may also apply to the cardiorenal patient (34) (Figure 6).

**Figure 7:**
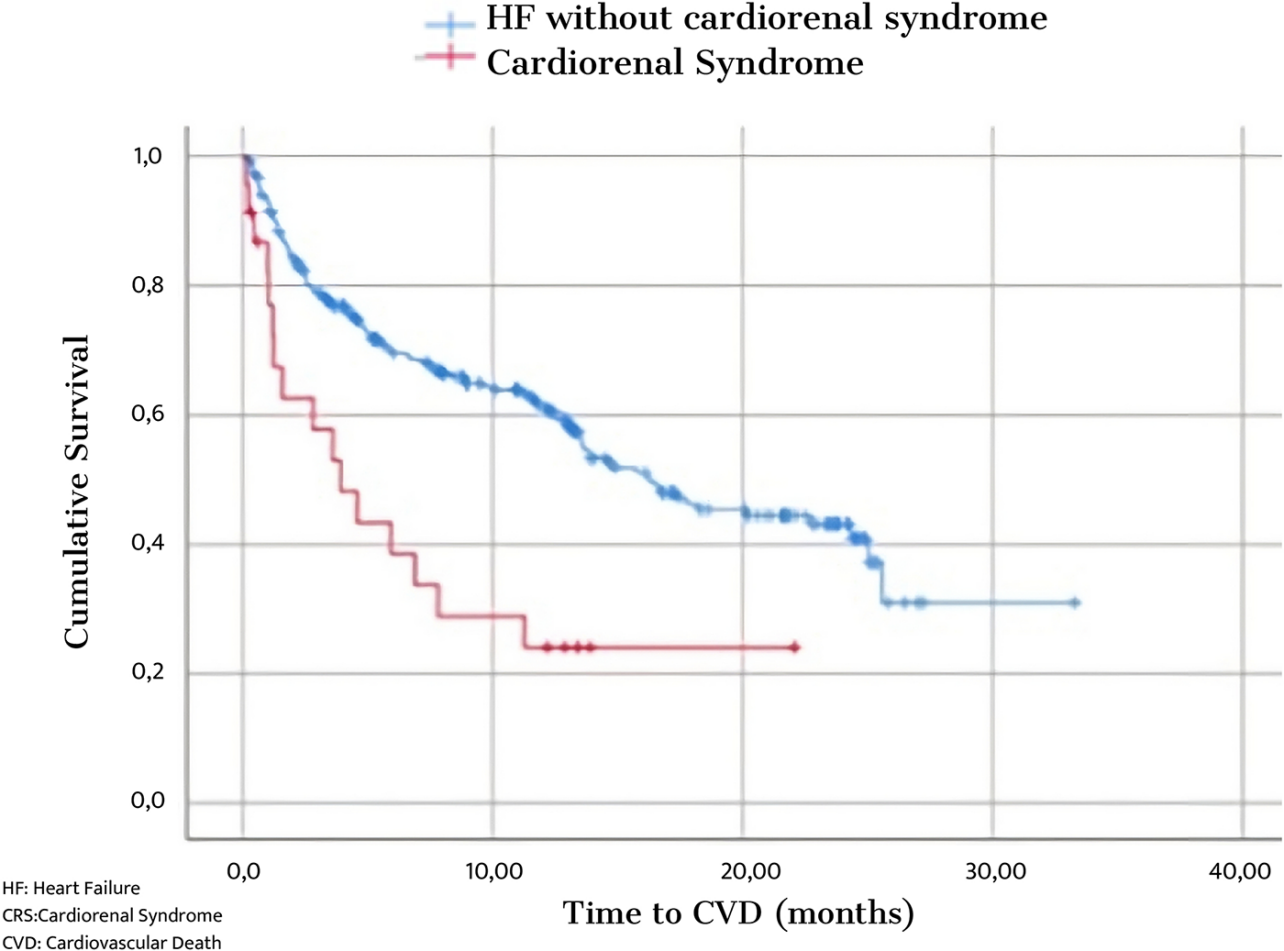
Survival in patients with Heart Failure compared to Cardiorenal Syndrome.

The NECPAL tool (see Supplementary Appendix) has been demonstrated to help identify the need to add PC in patients with complex chronic conditions, including HF and ACKD (36). Additionally, the PROFUND index has outperformed clinical prediction in predicting 12-month mortality in hospitalized patients with multiple comorbidities (37). Various prognostic factors have been described in ACKD and CHF, some of which are general survival criteria standard to other progressive chronic diseases and others specific to the disease that indicate the need for palliative support (38). CRS is associated with a worse prognosis, with a doubling of the risk of HF-related events and death from cardiac or renal causes compared with isolated HF (39) [Figure 7 (40)].

**Figure 8:**
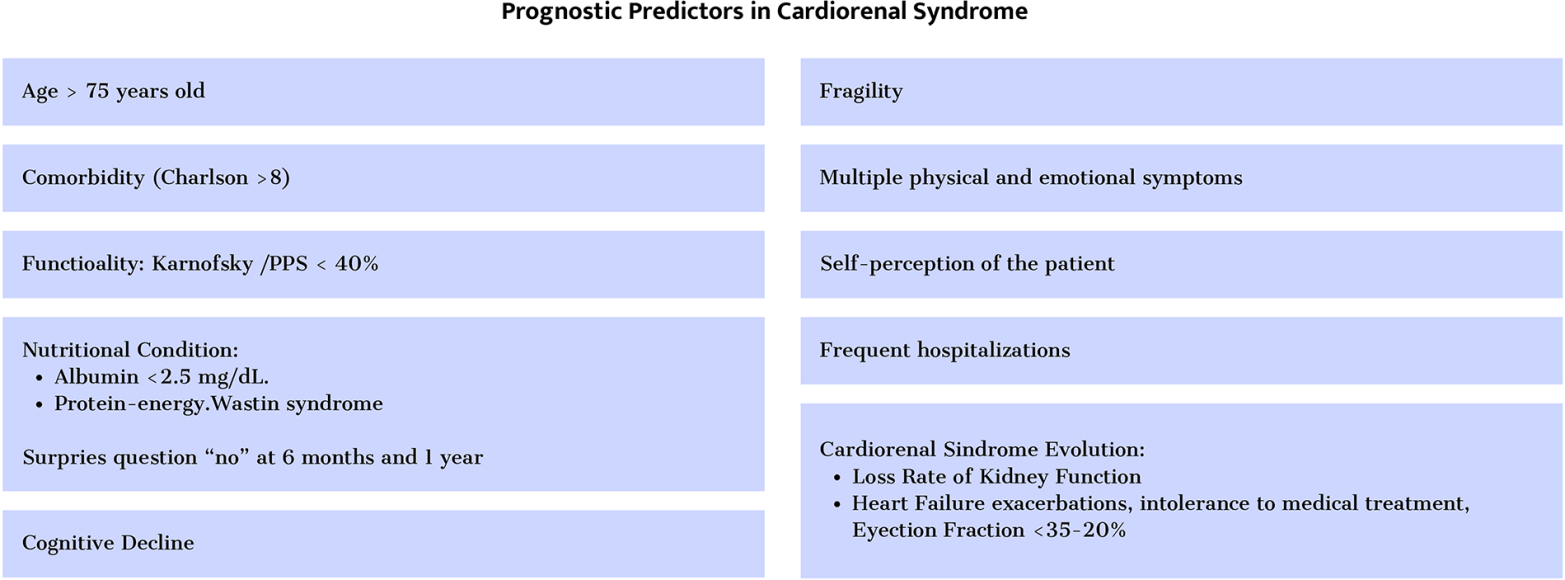
Prognostic predictors in cardiorenal syndrome.

The most used is the survival estimation by answering “no” to the surprise question as to whether we would be surprised if the patient died at six months or one year. The presence of geriatric syndromes, the number of admissions, and frailty are also markers of poor prognosis. The presence of multiple symptoms and their intensity also have prognostic value (41) (Figure 8).

**Figure 9:**
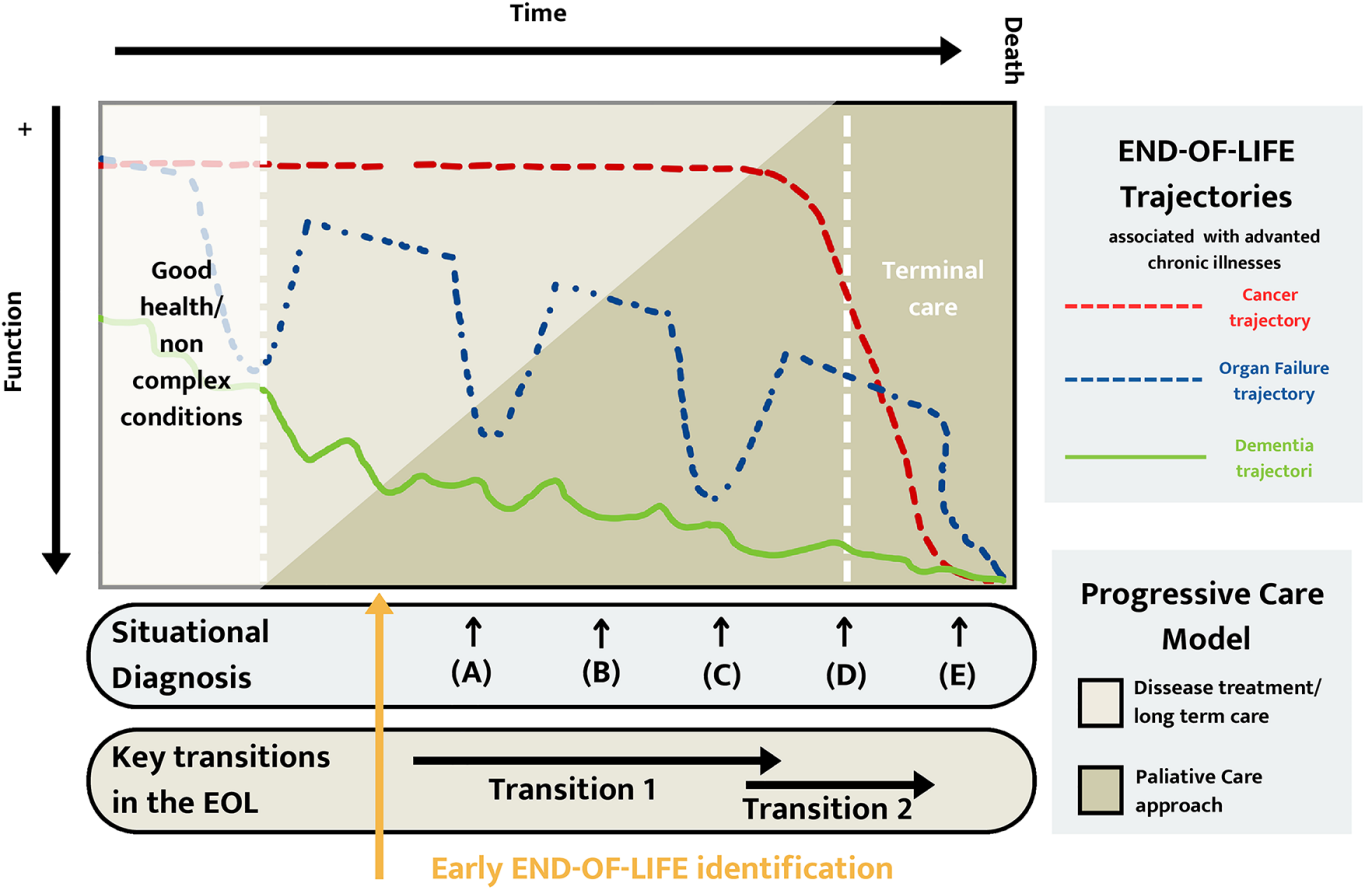
Functional end-of-life trajectory.

Knowledge of cardiorenal prognosis and functional trajectory will help identify disease progression, the need for PC, and adapting the therapeutic effort in end-of-life situations (42) (Figure 9).

## 4. Shared care planning

SCP is a structured and relational process developed by the Spanish Planning Association for Care (AEPCA) that aims to facilitate reflection and understanding among those involved in the care of an individual facing a disease trajectory. The process focuses on the person's care preferences and expectations, intending to promote shared decision-making in the current context and future care challenges, particularly in cases where the person cannot decide for themselves. SCP has evolved from previous terms and practices, such as living wills, advanced directives documents, and care planning communication processes.

AEPCA proposes a SCP because it is a more accurate concept and reflects the three areas of the process:

(a) The development of a care plan,
(b) The shared deliberation among those involved in the disease process (patient, family, and healthcare professionals),
(c) The comprehensive vision of care beyond purely biological terms.

All this information should be registered in the patient's medical record so that it can be consulted by all the professionals involved in the care process. Documentation of the shared care plan is an essential step representing a real challenge for the professional. It is advisable to have some section in the patient's clinical history where information obtained during the process is reflected (43–45).

SCP should meet the following requirements:

• Be objective, reflecting the patient's consensed decisions and avoiding the professional's subjective opinion.
• Reflect on the patient's values, expectations, beliefs, and desires.
• Identify the person delegated by the patient as the representative who will make decisions if the patient cannot do so.
• The patient himself should validate the information.

A document that captures advanced support decisions, including aspects related to renal and cardiac failure, can form a part of SCP. Additionally, SCP may also encompass other general aspects, such as life support, patients' preferences about hospitalization, pain management, and specific treatments that they may need in the future, such as implantable defibrillators for congestive heart failure, tube feeds for dementia, dialysis for renal failure, and ventilation for respiratory diseases. Clinicians are advised to familiarize themselves with the laws governing such documents in their practice areas (46).

Cardiorenal disease is a growing condition associated with a high burden of symptoms and comorbidities. A cardiorenal patient approaching the end of life copes with scenarios of great uncertainty about prognosis. On the one hand, a relatively stable slow deterioration. However, on the other hand, it is not possible to anticipate or predict when a new exacerbation or crisis will take place (Figure 9). These particularities require healthcare professionals to help patients tolerate uncertainty, which is a critical element of any care planning process through adequate communication skills.

The shared decision-making process in CRS is complex, and it is not acceptable to delay the expression of the patient's wishes during exacerbations, such as when they are hospitalized. It is essential to involve the patient early on and throughout the advanced disease. For example, choosing Kidney replacement therapy (KRT) depends more on the nephrologist than the patient. Therefore, it is crucial to support patients in the decision-making process, particularly when choosing between aggressive and conservative treatments (47). KRT may be used differently in cardiorenal patients compared to those with CKD, as it is often indicated for refractory vascular congestion and may be started earlier and with lower doses. Patients should still be informed about their KRT options, considering factors such as advanced HF, age, and frailty. Advanced HF therapies, such as percutaneous interventions or resynchronization therapy, may improve quality of life, but end-of-life care should focus on symptom relief and comfort. Limitations of treatments, including defibrillation and admission in the intensive care unit, should be discussed in advance (48).

## 5. Clinical management of the palliative cardiorenal patient trajectory

### 5.1. Indications and adjustment of cardiorenal protective drugs

**Figure 10:**
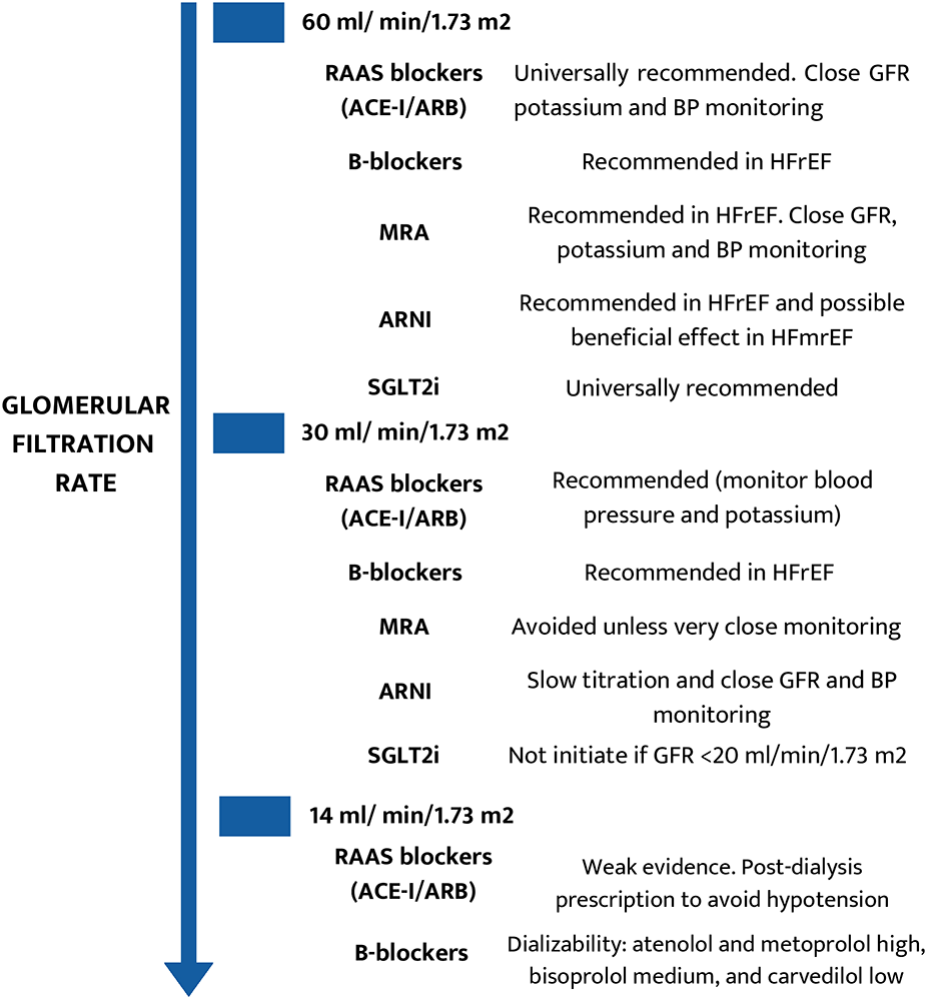
Drug recommendations in the different stages of chronic kidney disease.

Although there is limited evidence, cardiorenal patients may benefit from traditional and modern guideline-recommended medication. However, when prescribing medications that may impact kidney hemodynamics or have increased accumulation and potential toxicity in the setting of kidney dysfunction, caution should be exercised. Renin-angiotensin-aldosterone system (RAAS) blockers and *β*-blockers are the most widely studied and used agents across the spectrum of CKD. Both can be used in patients on dialysis and those who are not, providing cardiovascular protection without an increased risk of adverse events (Figure 10). With regards to RAAS inhibitors, hyperkalemia is a well-known limitation in their prescription. New potassium-binding agents such as patiromer and zirconium cyclosilicate can reduce the risk of hyperkalemia and allow for more liberal use, preventing the need for dose reduction, suspending medications, and maintaining their benefits (49–51).

On the other hand, β-blockers should be included in the armamentarium of cardiorenal patients, especially in cases of heart failure with reduced ejection fraction (HFrEF) and chronic coronary syndromes. In patients requiring renal replacement therapies, the use of β-blockers should be based on dialysis treatment for adequate chronotherapy (i.e., pre- or post-dialysis session), [Figure 10 (52)].

Mineralocorticoid receptor antagonists (MRA) have demonstrated their protective effect in cardiorenal patients with solid evidence until glomerular filtration rate (GFR) 30 ml/min/1.73 m^2^ (Figure 10). However, below GFR 30 ml/min/1.73 m^2^, MRA should only be used in selected cases with strict control of renal function, potassium levels, and blood pressure. Similar precautions to those taken with RAAS blockers should be observed, mainly when combined. While non-steroidal MRAs such as finerenone have demonstrated a safer profile in diabetic kidney disease, there is still a lack of solid evidence endorsing their safety and efficacy in cardiorenal patients, as noted in Figure 10. New potassium binders can also reduce MRA-associated hyperkalemia, making their use safer (50).

Two novel therapeutic groups have recently emerged offering new tools for reducing residual renal and cardiac risk in non-dialysis CKD patients: angiotensin receptor–neprilysin inhibitors (ARNI) and sodium–glucose cotransporter two inhibitors (SGLT2i). Sacubitril/valsartan has strongly demonstrated a consistent reduction in cardiovascular events in patients with CKD (up to GFR 30 ml/min/1.73 m^2^) and HFrEF (Figure 10) (50). The indication of ARNI could be extended to HF with midrange ejection fraction (HFmrEF) and, with caution, to patients with advanced CKD (GFR 15–30 ml/min/1.73 m^2^) (51).

SGLT2i (empagliflozin, canagliflozin, and dapagliflozin) has established a new era in cardiorenal patient management. These agents have demonstrated a reduction in major adverse cardiovascular events (MACE) and major adverse kidney events (MAKE) in cohorts with and without diabetes, the whole spectrum of patients with HF (irrespective of left ventricular ejection fraction), and CKD (up to GFR 20 ml/min/1.73 m^2^) (Figure 10). In addition, iSGLT2 shows an excellent safety profile with very low adverse events (primarily genital and mild urinary tract infections) (50). Although the GFR limits their prescription, once started, they can be continued until renal replacement therapy is initiated. It should be noted that SGLT2i could present an initial and mild reduction in GFR that is not associated with adverse clinical events (53). Thus, this initial GFR decline should be comprehensively evaluated, avoiding withdrawal in most cases.

### 5.2. How to deprescribe treatments?

Deciding whether to prescribe medications for palliative care patients with cardiorenal disease is a clinical challenge due to the variable clinical course of the disease and the modest accuracy of mortality prediction models (54).

The advanced cardiorenal disease is characterized by a progressive decline in functional class and a gradual increase in the burden of symptoms that include pain, anxiety, depression, sleep disturbance, fatigue, and dyspnea (55).

While there are no defined guidelines for stopping medical treatments, it is essential to prioritize and discuss transitioning to symptom relief and improving the patient's comfort with the patient. In patients with severe functional or cognitive impairment and end-stage irreversible disease, medication withdrawal should be considered when the risks or futility outweigh the benefits, monitoring or administering the medication is challenging, or the patient's drug adherence is complicated (56). Guideline-recommended medical therapies such as ARNI and SGLT2i have improved dyspnea and quality of life (57). Thus, these therapies and others addressing symptom burden should be continued if well-tolerated (58). Potassium-binding agents like patiromer and zirconium cyclosilicate can counteract the risk of hyperkalemia and allow the use of RAAS blockade drugs (59). Beta-blockers can control tachycardia and angina, and digoxin may be considered for patients with atrial fibrillation and high heart rates if beta-blockers are stopped or reduced (60, 61). However, digoxin should be used cautiously or avoided in patients with decreased renal function (62). Regular review of medical therapy and monitoring for potential side effects such as hypotension or worsening kidney function is crucial. Dose reduction or discontinuation of therapies should be assessed individually (63).

On the other hand, congestion is a significant contributor to the development and worsening of dyspnea (64). Therefore, diuretic treatment and adjustment should be a therapeutic goal in all the phases of the disease. In advanced cardiorenal patients, sometimes higher doses of diuretics are required, and often in combination, for example, with a thiazide-like diuretic, metolazone, and carbonic anhydrase inhibitor, acetazolamide can be added to potentiate the natriuretic effects of loop diuretics (65).

Finally, re-adjustment or prescribing back therapies should be considered if the patient's clinical status improves.

## 6. Symptom management

**Figure 11:**
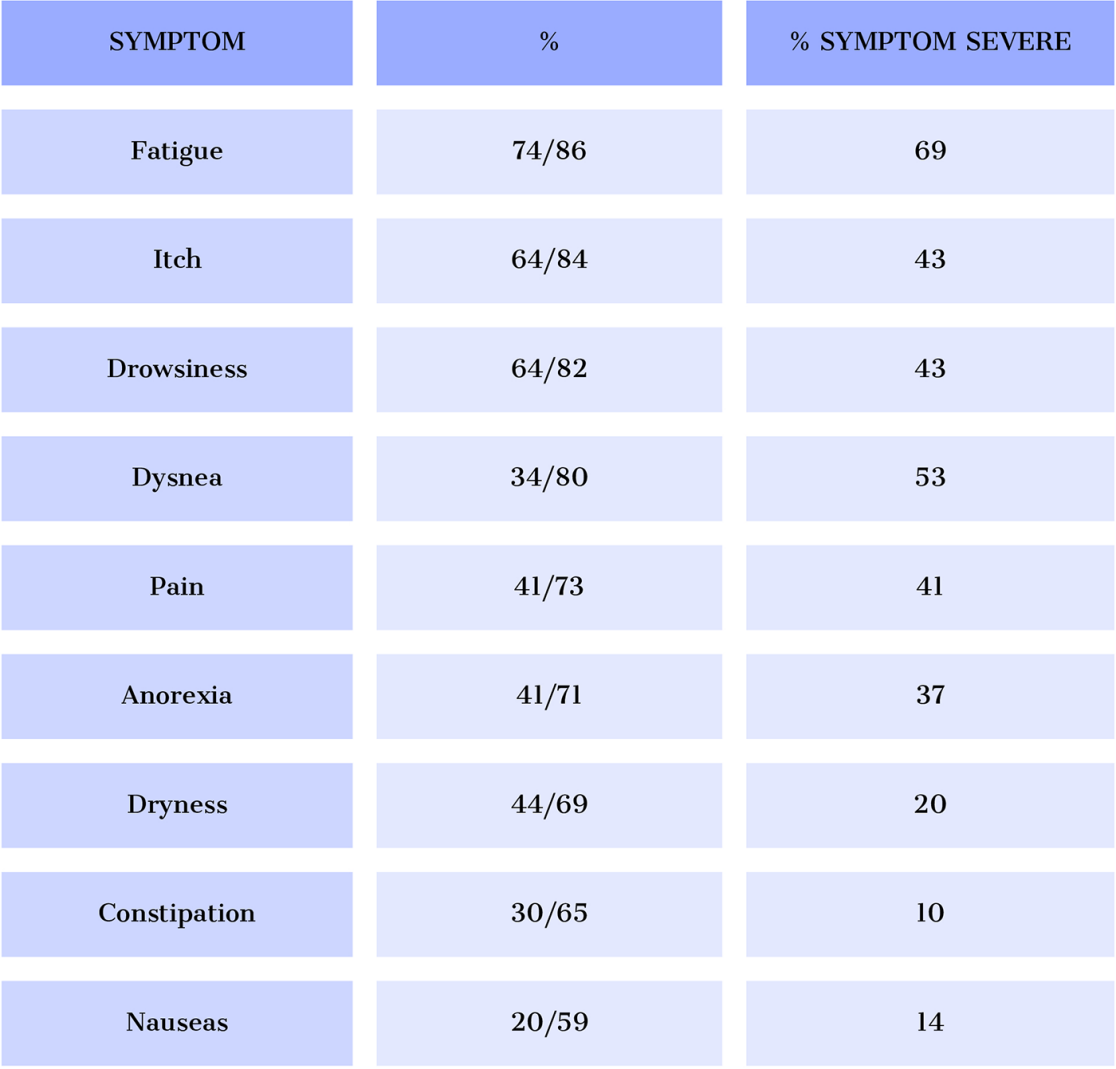
Prevalence of the most frequent symptoms and degree of severity in patients with advanced cardiorenal disease.

Advanced cardiorenal patients often experience a symptom burden (66–68) comparable to that observed in patients with advanced cancer and other life-limiting illnesses (Figure 11) (66, 67)) (68, 69). Unfortunately, however, physicians often neglect many of these symptoms and are unaware of their relevance to patients’ quality of life. Therefore, regular and holistic symptom assessment should be integral to any palliative care program.

**Figure 12:**
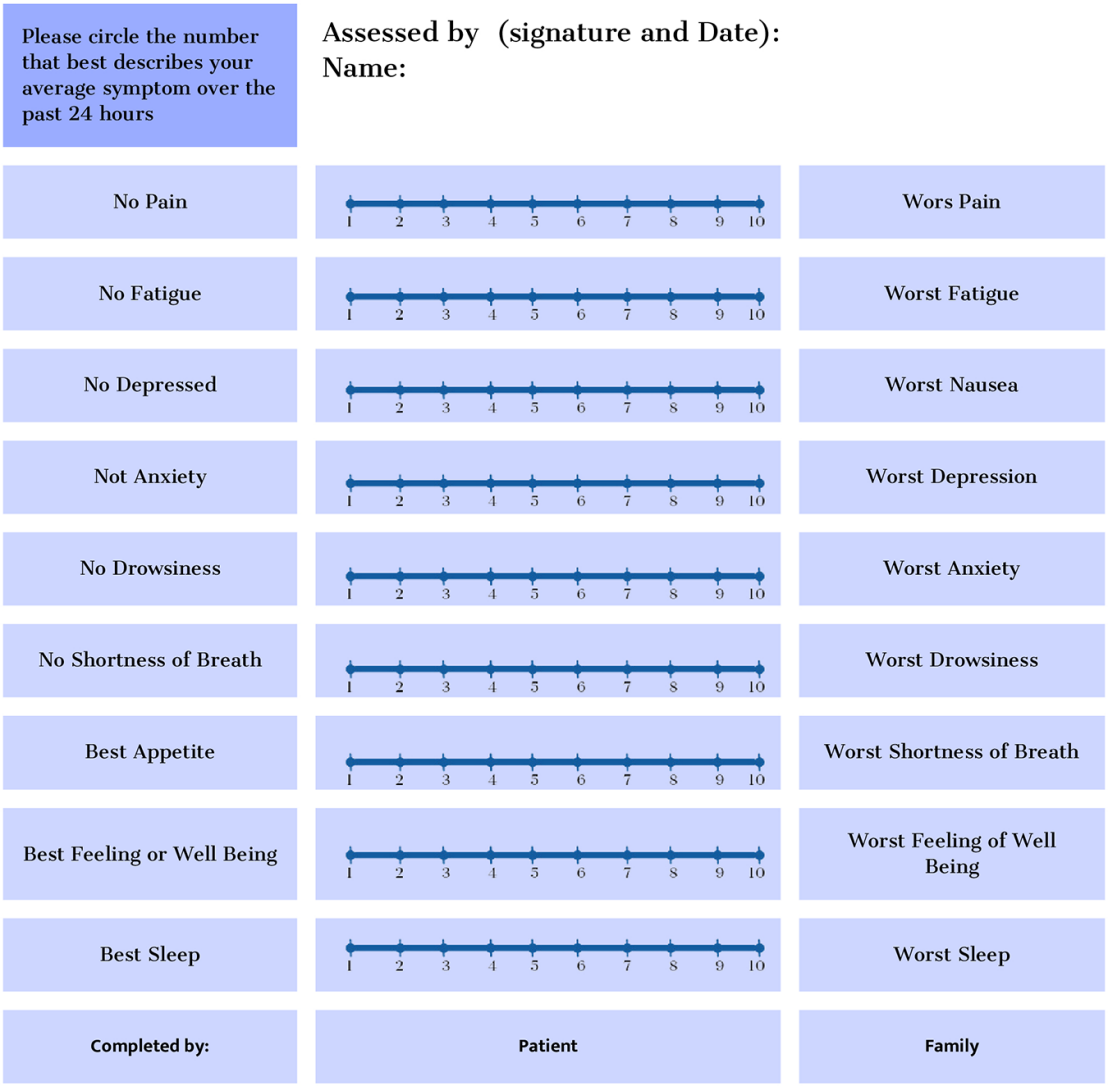
Edmonton Symptom Assessment System (ESAS-r).

**Figure 13:**
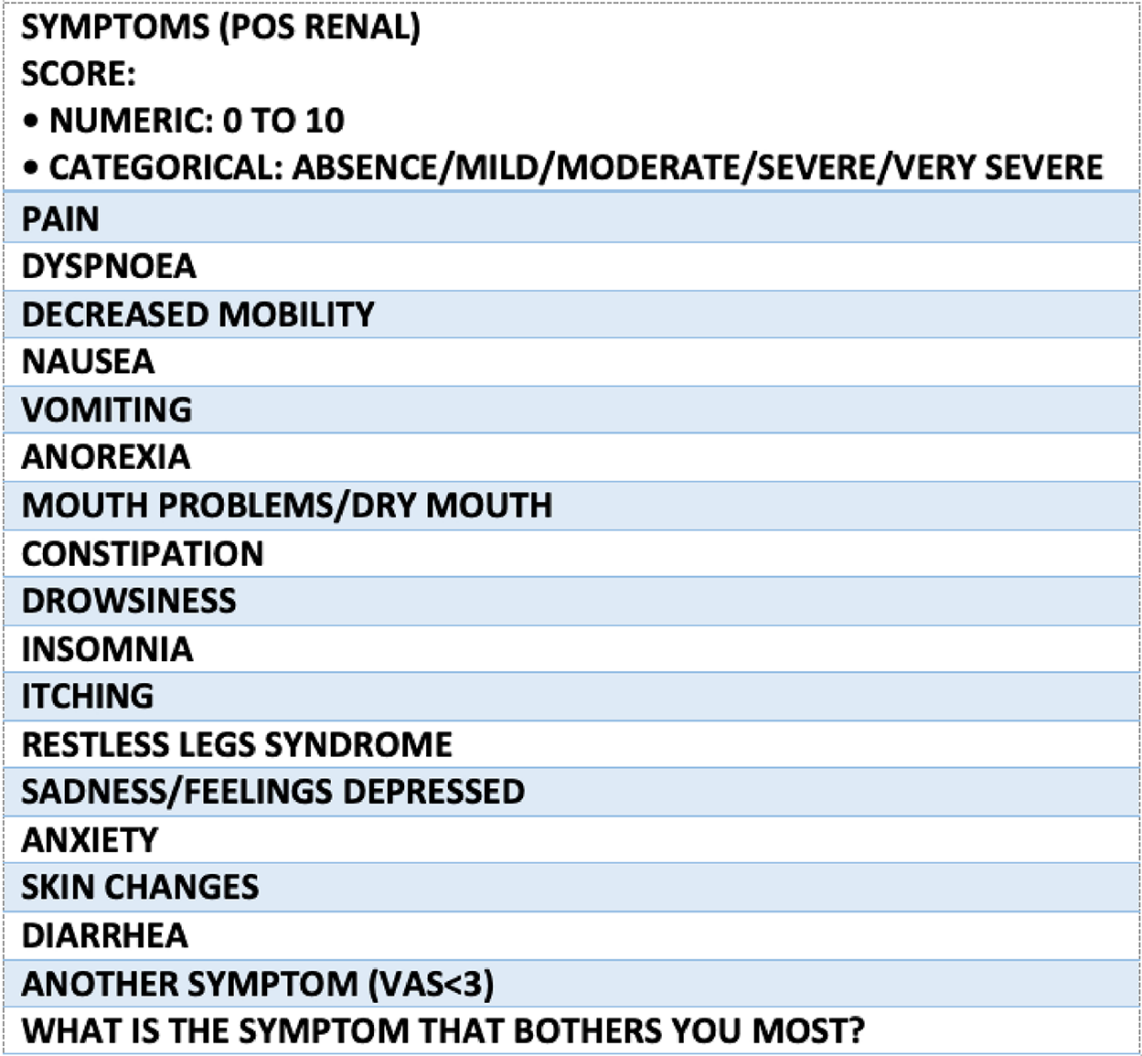
Integrated palliative care outcome scale (IPOS-renal).

Use validated tools and multidimensional instruments to assess symptoms in advanced cardiorenal patients. The Edmonton Symptom Assessment System (ESAS) is the most common tool. At the same time, the Palliative Care Outcome Scale-Symptoms Renal (POS-S Renal) is more specific to end-stage renal disease patients. Both assess the prevalence and intensity of symptoms, but subjective patient evaluation is necessary to evaluate their impact on quality of life. Advanced cardiorenal patients may have multiple concurrent symptoms, so symptom clustering is recommended to adopt a holistic approach based on the patient's needs (Figure 12 (70) and Figure 13 (71)) (71, 72).

### 6.1. Astenia, anorexia, and cachexia

**Figure 14:**
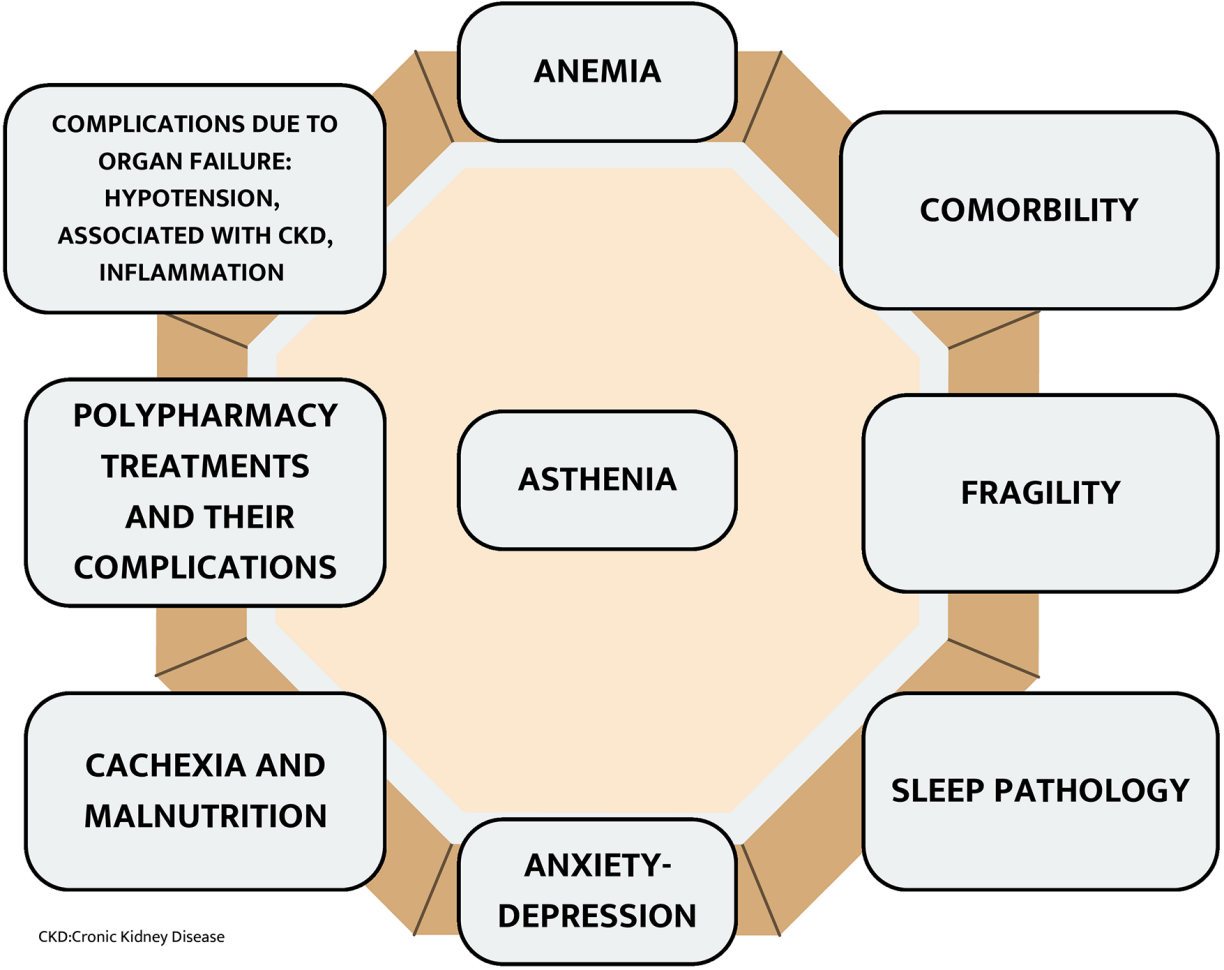
Etiopatogenic factor of asthenia.

Advanced cardiorenal patients often experience a cluster of symptoms, including asthenia, anorexia, and cachexia, which significantly impact their quality of life (66). Asthenia (Figure 14), a persistent feeling of fatigue and weakness, can be treated with non-pharmacological strategies such as exercise, sleep hygiene, and nutritional advice. In some cases, psychostimulants and nutritional supplements may be beneficial (73).

### 6.2. Pain

Around 60% of CKD patients suffer from pain, with increasing prevalence and intensity as the disease progresses (66–68). Pain has a significant impact on physical and mental quality of life, interfering with function, and is often undertreated due to incorrect assessment. The WHO analgesic ladder is a practical management approach, using paracetamol in the first step, tramadol in the second, and fentanyl, methadone, and buprenorphine in the third (74–76). In general, opioids with active metabolites that are excreted via the kidneys are not recommended, with a GFR of less than 30 ml/mn. Mainly including morphine, oxycodone, and hydromorphone, although there is much less data on the latter two (76–81).

Morphine clearance is similar in patients with altered and normal kidney function. However, some glucuronide metabolites significantly accumulate in patients with reduced kidney function. They may contribute to CNS depression, sedation, and severe and prolonged respiratory depression, which may be delayed in presentation (82).

There are no specific dose adjustments provided in the manufacturer's labeling. However, it is possible to find some recommendations on morphine dosing according to renal creatinine clearance (CrCl). For example, CrCl ≥60 ml/min: No dosage adjustment necessary; CrCl 30–<60 ml/min: Consider using an alternative opioid analgesic. If necessary, administer 50%–75% of the usual initial dose; also consider extending the dose interval. Titrate cautiously to response; CrCl 15–<30 ml/min: Avoid use. If necessary, administer 25%–50% of the usual initial dose; may also consider extending the dose interval. Titrate cautiously to response; CrCl <15 ml/min: Avoid use (83).

### 6.3. Pruritus

Pruritus, an unpleasant sensation that causes the desire to scratch, is common in advanced cardiorenal disease, affecting up to 40%–50% of patients (66, 67). Its multifactorial etiopathogenesis needs to be more adequately evaluated, leading to undertreatment. Treatment for uremic pruritus involves skin hydration as the primary measure. Additional therapies that have demonstrated efficacy include low-dose gabapentin and pregabalin, selective serotonin reuptake inhibitors, low-dose heterocyclic antidepressants, and opioid antagonists (84, 85).

### 6.4. Anxiety and depression

Anxiety and depression are common in cardiorenal patients (66, 67). Depression is particularly prevalent and can lead to poor self-care and therapeutic adherence. Treatment for depression requires a multidisciplinary approach, including antidepressants, cognitive-behavioral therapy, or exercise training. SSRIs like fluoxetine and paroxetine are considered the safest and first-line choice if medication is necessary, while other options like trazodone and mirtazapine may also be used (86).

### 6.5. Insomnia and restless leg syndrome

Insomnia is persistent difficulty initiating or maintaining sleep, accompanied by significant distress or impairment in functioning. Adequate sleep is essential for metabolic, endocrine, and immune system functioning. Cardiorenal patients often have sleep disorders and accompanying symptoms. Treatment involves addressing underlying factors, sleep hygiene, and medication (66–68). Restless leg syndrome (RLS) is a sensory-motor disorder characterized by uncomfortable sensations in the lower limbs during the night that are relieved by movement. Its prevalence in chronic kidney disease patients is up to 30%. Non-pharmacological approaches like cold dialysis, exercise, and physiotherapy have been effective in managing RLS, along with gabapentin as the preferred pharmacological treatment (87).

### 6.6. Dyspnea

Shortness of breath is common in advanced stages of cardiorenal disease, and its intensity increases as the disease progresses. Dyspnea may be related to various factors, such as myopathy, sarcopenia, or frailty. However, it is often secondary to pulmonary congestion or low output caused by ventricular and/or valvular dysfunction. Maintaining drugs with cardiorenal benefits is vital to maintaining hemodynamic stability, achieving euvolemia, and decreasing venous congestion. In refractory dyspnea, opioids and home oxygen may be necessary during the final phase of the disease. Opioids improve dyspnea by acting on its central perception, achieving a decrease in metabolic rate and ventilation requirements. Its role in the control of dyspnea has been widely evaluated in the literature, with morphine being the most studied opioid and with the greatest experience of use, especially orally and subcutaneously. However, morphine (as explained above) (88) is not ideal due to the accumulation of active metabolites in renal failure with a higher risk of neurotoxicity (89). Alternative options should be proposed, considering first intranasal or subcutaneous fentanyl 100–200 mcg/4–6 h based on recent studies that include heart failure population, still limited for GFR under 15 ml/min. Evidence of absence or minimal neurotoxicity with these doses in scenarios like withdrawing dialysis suggests the potential benefit and absence of harm for these populations. However, there is still limited data (90–92). For opioids such as oxycodone, hydromorphone is still higher.

## 7. Advanced measures in cardiorenal patients

• Hemodialysis

It should only be considered in patients with heart failure (HF) and stage 5 chronic kidney disease (CKD) to relieve uremic and/or congestive symptoms, considering the unfavorable impact on the cardiovascular system, need for venous access, displacement to a healthcare center, and when the patient does not accept conservative or palliative treatment.

• Continuous ultrafiltration techniques

It is indicated in situations of acute or decompensated HF that is refractory to diuretic treatment despite not having shown advantages in terms of renal function evolution or mortality. Isolated ultrafiltration can reduce hospitalization days and weight compared to diuretic therapy (93).

Peritoneal dialysis (PD) is indicated in patients with the following characteristics:

(a) Advanced heart failure with optimal treatment (94),
(b) Frequent decompensations in systemic congestion (95). Multiparametric assessment should be performed to determine congestion phenotype: clinical, biomarkers, VExUS (venous excess ultrasonography score),
(c) Diuretic resistance is defined as attenuation of maximal diuretic effect resulting in reduced natriuresis and diuresis, limiting the possibility of achieving euvolemia (96),
(d) CKD stage 3–4.
(e) There are no contraindications for PD (97), and there is support for performing the technique if necessary.
• Removal of the renal replacement therapy

Since the goal of using these techniques in palliative care for cardiorenal patients is to improve symptoms and quality of life, they should be suspended when these goals are not met, especially when there are technical or logistical difficulties in performing the technique, situations of cardiogenic shock, deterioration of frailty, cognitive decline, worsening of quality of life, or the patient's desire.

• Implantable cardioverter defibrillator (ICD)

In the patient with ACKD, the indication for an ICD should be carefully evaluated, as study results suggest that chronic kidney disease may negatively affect the efficacy and safety of the ICD, leading to a higher number of unnecessary ICDs shocks (98) and more electrode complications (fractures, pacing losses, others) (99). ACKD itself significantly decreased the event-free survival rate in heart failure patients who received an ICD (98) and lowered the therapeutic efficacy of the ICD compared to patients without ACKD.

On the other hand, in the late stages of the disease, especially in the last days of life, the ICD may shock, causing pain, causing unpleasantness for the patient and family, and prolonging life unnecessarily. There is broad consensus on the desirability of deactivating these treatments. Anti-tachycardia therapies can be deactivated by maintaining the “monitoring only” mode.

• Levosimendan

It was associated with a lower incidence of cardiorenal syndrome and better recovery of renal function at discharge, so Levosimendan appears to have some renoprotective effect and may be an effective and safe option in patients with advanced chronic kidney disease and heart failure. However, it is eliminated mainly by the kidneys, so the dose may need to be adjusted in these patients to avoid adverse effects and accumulation (100, 101).

A meta-analysis compared mortality in 4 studies with a total of 4,458 patients; Levosimendan significantly reduced mortality compared with the use of a control (OR: 0.62; 95% CI: 0.46–0.84), milrinone (OR: 0.50; 95% CI: 0.30–0.84) or dobutamine (OR: 0.75; 95% CI: 0.57–0.97).

The pooled analysis of 8 studies found that levosimendan was associated with a significant increase in GFR compared with dopamine use (SMD: 1.46; 95% CI: 0.88–2.03) or control (SMD: 1.67; 95% CI: 1.17–2.18). Furthermore, dobutamine use significantly elevated GFR compared with dopamine use (SMD: 1.28; 95% CI: 0.59–1.96) or control (SMD: 1.49; 95% CI: 0.87–2.12), as well as with dobutamine use (SMD: 1.46; 95% CI: 0.88–2.03).

Regarding serum creatinine values, five reported trials (599 patients) indicated that the use of levosimendan [SMD: −0.58; 95% CI: -(0.9–0.23)] or dobutamine [SMD: −0.54; 95% CI: -(1.07–0.01)] significantly decreased serum creatinine compared to placebo. Therefore, this Meta-Analysis of Randomized Controlled Trials levosimendan had the highest *P*-score, indicating that it most effectively reduced mortality and improved renal function, even in patients with renal failure (102).

• Dobutamine

Studies are comparing Levosimendan with Dobutamine. In a Portuguese study of 108 HF patients, the incidence of CRS was higher in the dobutamine group, and they more often had incomplete recovery of renal function at discharge (100).

• Transcatheter aortic valve replacement (TAVR)

Several meta-analyses (103) found TAVR to be a viable option for patients with ACKD and aortic valve disease, albeit with higher adverse event rates. European multicenter studies (104) also found TAVI to be a helpful option but with higher rates of renal and cardiac complications. These studies suggest that TAVR is a helpful option for patients with severe symptomatic aortic stenosis and ACKD but requires careful monitoring and appropriate care planning to avoid complications.

• The artificial heart (TAH)

The total artificial heart is an implanted device approved to stabilize patients who are often critically ill, which is why decision-making for a TAH is complex. There are ethical implications given the prognostic uncertainties. An article published this year lists four critical areas: the decision maker, the minimum acceptable outcome/maximum acceptable burden, living with the device, and dying with the device.

Some advice is as follows: (1) maximize conversations with patients and their decision-makers; (2) social support is critical; (3) identify legal decision-makers; (4) preparedness planning, including discussions about end-of-life care and discontinuation of treatment, is crucial (105)

## 8. Approach to the last days situation of the palliative cardiorenal patient

### 8.1. Understanding integrated care

Whole-person care is essential for approaching ethically and clinically proper care during the last days of life for cardiorenal patients. Quality of care in this phase is highly determinant for the disease course. Poor quality of the dying process could take down the efforts experienced through the disease and therapeutic trajectory. No one can die again; it means we do not have a second chance for better care. The health care team and relatives have one opportunity to bring the best care in this profoundly human scenario, which should be understood more as a biographical than a clinical event (106).

### 8.2. Identifying the right moment

The last days of life precede death and require specialized care emphasizing comfort and essential care. Recognizing this phase is challenging, particularly in hospitals focused on curative processes (107). When dyspnea or other symptoms are managed by dialysis, discontinuation may be appropriate. This change in therapeutic approach should be understood as one step in the overall therapeutic effort. Focusing on the human being and less on the dialysis technique can improve the quality of care (108).

It is described as a series of clinical signs of agony whose presence can predict death in the next few days. Mind and Hufkens evaluated eight criteria (107):

(1) White cold nose
(2) Cold extremities
(3) Lividness
(4) Cyanotic lips
(5) Sleepiness > 15 h/d
(6) Rattles
(7) Apnea pauses > 15″
(8) Anuria <300 ml/d

The protocol Intro-PAC-WDC (90, 108) describes how to bring support for clinicians when withdrawal dialysis (WD) is needed. It focuses step by step on dealing with information for patients and relatives, teamwork regarding WD, bioethical considerations in every step, and adjustments of the therapeutic approach in the limiting therapeutic effort journey, among many other topics. It may be relevant for cardiorenal patients under dialysis.

### 8.3. Controlling symptoms

**Figure 15:**
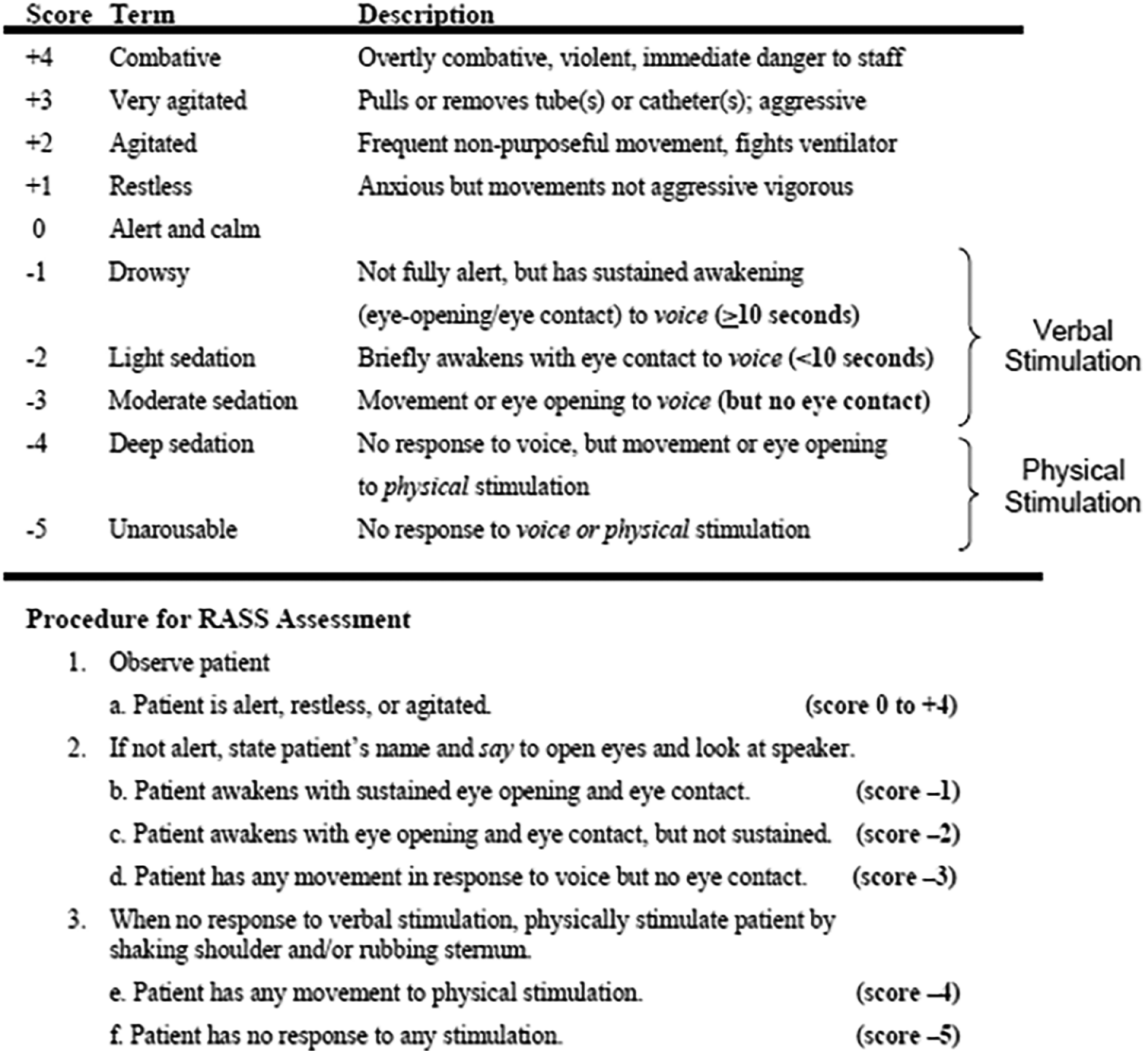
Richmond Agitation Sedation Scale (RASS).

The three most common symptoms in the last days of life are dyspnea, delirium, and pain (66, 109). Pain in the cardiorenal population is expected to be related to low cardiac output. Intranasal fentanyl has been shown to effectively control pain during the withdrawal of dialysis in a cohort of seven patients without signs of opioid-induced neurotoxicity (110). Midazolam may be prescribed in low doses for delirium, adjusted according to the Richmond Agitation Scale for Sedation (111, 112) [Figure 15 (111, 112)]. Palliative sedation therapy may be prescribed for refractory symptoms, but ethical authorization and selecting the right drug and doses are crucial. Keeping the family informed about drug prescriptions can help them understand and adjust to the end-of-life process (90).

### 8.4. Whole person care

It is essential to understand human and clinical suffering from an anthropological perspective. Clinicians who are not prepared to take care of suffering in clinical settings may act under one of these 3F: “flying,” avoiding the clinical scenario and providing poor quality of care; “frozen,” getting paralyzed and not running a decision-making process to provide the best care or “fighting” therapeutically and ordering diagnostic procedures or interventions not focused in the needed care (113). Being present and compassion are two essential elements clinicians should learn about to help the best care practice when the cardiorenal disease journey is approaching its end (114).

## 9. Discussion

There are scarce documents on palliative care in cardiorenal patients in world literature. This is a consensus document and does not provide new results. However, the novelty of the document is the concept of palliative care in cardiorenal patients, which has been barely covered in the literature. Another interesting topic is SCP. This paper gathers the current knowledge on the management of patients with CRS who require PC and provides practical guidelines to improve the care and treatment of these patients. In our opinion, the manuscript fills a gap in the scientific literature by providing a comprehensive and consensus-based approach to the palliative management of cardiorenal patients. Its findings contribute significantly to progress in the research discipline by providing clear, evidence-based guidelines for clinicians, thereby improving the care and quality of life of cardiorenal patients worldwide.

### 9.1. Limitations

Patients with ACKD are not included in HF clinical trials. Furthermore, it is difficult to find funding to carry out clinical trials on symptomatic treatment adapted to cardiorenal patients, and sometimes ethical limitations may be found. For this reason, most of the recommendations are expert opinions. There is an evident need for further research to assess the efficacy of such care in areas such as symptom improvement, hospitalization reduction, impact on caregivers, and cost-effectiveness. This review is a promising foundation for future investigations in this crucial domain.

### 9.2. Future directions

The writing group endorses the need for a dedicated palliative cardiorenal interdisciplinary team that leads the early identification of cardiorenal patients who need PC and jointly manages appropriate clinical interventions across the inpatient and outpatient settings. This collaboration would also supervise cross-training among nephrology, cardiology, palliative care fellows, and nursing to foster a deeper understanding of the needs. This group also considers it mandatory to include patients with ACKD in clinical trials of drugs or devices indicated for HF. The results of clinical trials on SGLT2i or ARNI in patients on dialysis are awaited, which may provide us with more evidence-based recommendations.

## 10. Conclusion

Patients with CRS constitute a population highly amenable to PC due to their elevated mortality rates, substantial symptom burden, and the imperative for prognosis-based decision-making and shared care planning.

Effective identification of CRS patients who require PC involves employing tools to assess frailty, prognosis, functionality, and symptoms. Clinical evaluation and the coordinated use of cardio-nephroprotective strategies tailored to each disease stage are crucial for managing congestion maintaining hemodynamic stability, and symptom control.

PD emerges as a viable option for symptom improvement in CRS patients with venous congestion and diuretic resistance. The use of ICD should be restricted during the final days of life, and Levosimendan may be considered in advanced stages.

Finally, it is mandatory to take an appropriate approach to the last days situation of the patient; a multidisciplinary approach is crucial to identifying the right moment, controlling symptoms (especially dyspnea) with drugs adapted to kidney failure, and prescribing a palliative sedation therapy if refractory symptoms. Being present and compassion are two essential elements when the cardiorenal disease journey is approaching its end.
